# The impact of the COVID-19 pandemic on children and adolescent mental health in-patient service use in England: interrupted time-series analysis of national patient records

**DOI:** 10.1192/bjo.2024.9

**Published:** 2024-03-21

**Authors:** Apostolos Tsiachristas, Josephine Holland, Boliang Guo, Prathiba Chitsabesan, Kapil Sayal, Anees Ahmed Abdul Pari

**Affiliations:** Nuffield Department of Primary Care Health Sciences & Department of Psychiatry, University of Oxford, UK; Mental Health and Clinical Neurosciences, Institute of Mental Health, University of Nottingham, UK; Pennine Care NHS Foundation Trust, Manchester, UK; NHS England, East of England, Cambridge, UK

**Keywords:** In-patient treatment, community mental health teams, COVID-19, pandemic, psychiatric hospital admissions

## Abstract

**Background:**

During the initial phases of the COVID-19 pandemic, children and young people (CYP) faced significant restrictions. The virus and mitigation approaches significantly impacted how health services could function and be safely delivered.

**Aims:**

To investigate the impact of COVID-19 lockdowns on CYP psychiatric admission trends during lockdown 1 (started 23 Mar 2020) and lockdown 2 (started 5 Nov 2020) of the COVID-19 pandemic in England.

**Method:**

Routinely collected, retrospective English administrative data regarding psychiatric hospital admissions, length of stay and patient demographic factors were analysed using an interrupted time series analysis (ITSA) to estimate the impact of COVID-19 lockdowns 1 and 2 on service use trends. We analysed data of 6250 CYP (up to 18 years of age) using ordinary least squares (OLS) regression analysis with Newey–West standard errors to handle autocorrelation and heteroscedasticity.

**Results:**

Psychiatric hospital admissions for CYP significantly fell during lockdown 1, and then fell even further during lockdown 2. A greater proportion of admissions during lockdown were out of area or to independent sector units. During lockdown, the average age of CYP admitted was higher, and a greater proportion were female. There was also a significant increase in the proportion of looked-after children and CYP from the most socioeconomically deprived areas admitted during lockdown 2.

**Conclusions:**

During both lockdowns, fewer CYP had psychiatric admissions. The subsequent rise in admissions for more socioeconomically deprived CYP and looked-after children suggests that these CYP may have been disproportionately affected by the pandemic, or overlooked during earlier phases.

During the COVID-19 pandemic in the UK, lockdowns, as well as other restrictions, were instigated to prevent the National Health Service (NHS) from becoming overwhelmed and to limit deaths.^1^ For children and young people (CYP), this caused a major interruption to in-person learning, social and community networks, recreational activities and ready access to healthcare.^2^ Although CYP were less likely to contract severe forms of COVID-19 requiring hospitalisation or to die from the infection,^3^ because of concerns about their ability to asymptomatically spread the virus to those more vulnerable, the restrictions placed upon CYP were as strict as, and in some ways stricter (e.g. school closures) than, those placed upon working-age adults.

The COVID-19 pandemic had profound mental health consequences for CYP.^2,4^ As well as the direct impact of the pandemic and its restrictions on mental health, COVID-19 also affected service delivery and availability for people with mental health disorders. Owing to concerns about the risk of spreading the virus, many out-patient mental health teams moved to remote working almost completely, with reduced access to many treatments and interventions. Although in-patient wards remained open, they faced significant challenges due to staff sickness or self-isolation, social distancing and patient infection outbreaks. Mental health services staffing was also further stretched as some healthcare resources were redeployed to deal with COVID-19 in general hospitals.

Since the start of the COVID-19 pandemic, there has been very limited research on the impacts on in-patient mental health services, with the focus more on adult community and out-patient populations.^5^

Out-of-area mental health admissions have caused significant controversy over recent years, with numerous media articles describing negative experiences, and policy initiatives aiming to minimise these occurrences.^6^ The impact of the pandemic on out-of-area admissions is not yet known. A better understanding of in-patient mental health services use during the pandemic is crucial to help plan effective service provision going forward in the pandemic recovery period, and to assist mental healthcare providers and commissioners to better respond to future disruptions.

This study therefore aims to investigate the impact of the early stages of the pandemic on in-patient mental health admissions for CYP, in terms of service use and out-of-area admissions. We hypothesised that the national lockdowns, introduced in England on 23 March 2020 and 5 November 2020, led to a reduction in CYP mental health admissions in England.

## Method

### Study design

NHS England, through seven regional specialised commissioning teams, directly commissions specialised CYP mental health in-patient provision.

In this observational retrospective study, we compared changes in the use of in-patient mental health services by CYP (up to 18 years of age) before and after the onset of the COVID-19 pandemic lockdowns in England. To do this, we adopted a quasi-experimental study design following guidance from the Medical Research Council on conducting natural experiments.^7^

### Data

The anonymised individual-level electronic medical records on CYP in-patient admissions were extracted from the national specialised mental health Patient Level Dataset across England between 1 January 2018 and 31 March 2021. The specialised mental health dataset was populated by provider NHS Trusts and held nationally in secure databases (this collection has now retired and is now merged with the Mental Health Services Data Set).

Commissioned in-patient services were provided by 41 NHS Trusts and 14 private hospitals. The data included the dates of hospital admission and discharge, type of ward and whether the admission was to a private psychiatric hospital. It also included sociodemographic characteristics of the admitted CYP, including age, gender, ethnicity, whether they had been looked after or were in full-time education, and socioeconomic deprivation based on the Office of National Statistics – Index of Multiple Deprivation (ONS-IMD). Ethnicity was used to create a dummy for identifying CYP from Black, Asian and Minority Ethnic (BAME) backgrounds.

Data were converted into weekly number/rates of mental hospital admissions, reflecting 169 weeks (117 weeks pre-pandemic and 52 weeks after pandemic onset). Out-of-area admissions were defined as admissions further than 50 miles from the individual's residence (NHSE report, 2014)^8^ or a clinician's notification of out-of-area admission based on natural clinical flows (i.e. acceptable clinical flow to units, recognising that there may be patient choice or specific clinical needs to admit outside). In terms of missing data, if the date of discharge was missing, we imputed length of stay with the mean within-patient length of stay. However, if a person only had one admission, the length of stay was treated as a missing observation. This approach was determined after performing a missing data analysis and looking at the distribution of missing observations across time. Moreover, we defined a variable as the average (entire) length of stay of all individuals admitted in a week. We also defined a variable based on the mean number of total admissions per person admitted in each week. This was to act as a proxy for the mental health of individuals admitted each week; it was expected that if individuals with greater mental health issues (i.e. those with multiple hospital admissions during the entire observation period) were admitted in a week, this variable would be higher.

### Statistical analysis

We performed an interrupted time-series analysis (ITSA) with a single group (i.e. no control group available) and two ‘events’ to estimate the immediate (i.e. in the first week of the event) and subsequent (from the second week until the last week of the event) impacts of the pandemic and associated public health measures. The first event occurred on 23 March 2020 when initial restrictions started being legally put into place, and the second event occurred on 5 November 2020 when the second wave lockdown started. ITSA models the impact of an event (in this case the lockdown, which was a public health measure to mitigate the COVID-19 pandemic) on a time-varying outcome. This approach is considered a strong quasi-experimental design and has been applied across a wide range of healthcare settings.^9^

Pre-existing time trends, immediate impact and subsequent impact were all assumed to be linear and were estimated with ordinary least squares (OLS) with Newey–West standard errors to handle autocorrelation in addition to possible heteroscedasticity. We used the Cumby–Huizinga test to ensure that each fitted ITSA model accounted for the correct autocorrelation structure (i.e. number of lags). As a sensitivity analysis, we also performed ITSA using generalised least squares in case the linear trends assumption was violated.

The ITSA was performed on a number of outcomes of interest, including weekly number of hospital admissions and length of stay, weekly rate of private hospital admissions, weekly number of out-of-area admissions, weekly number of admissions by patient characteristics (i.e. looked-after status, BAME background and ONS-IMD quintiles (with 1[th]=[th]most deprived to 5[th]=[th]least deprived).

To estimate the impact of the pandemic on hospital length of stay, inverse probability weighting (IPW) was used to adjust for changes in the composition of the sample (i.e. differences in the types of CYP seen by services before and after the pandemic onset). Logistic regression was performed to estimate the propensity of a weekly observation occurring after the first lockdown (compared with before it) based on the mean age, proportion of females, proportion of CYP in full education, proportion of looked-after children, proportion from BAME backgrounds, proportion of highest quintile of ONS-IMD, mean number of hospital admissions per person and proportion of individuals with censored data. The latter variable was included in the propensity score as a dummy indicating whether individuals were admitted right at the end of the study's observation period and they had no discharge date (i.e. to account for right censoring). The observations used in the ITSA were weighted based on the inverse probability of being observed after the first lockdown, adjusting therefore for differences in the patient case mix before and after the first lockdown. Following good statistical practice, we also used the confounding variables (i.e. those used in the logit regression to estimate the propensity score) in the ITSA as covariates.^10,11^

## Results

### Patient characteristics

Between 1 January 2018 and 31 March 2021 there were 10 657 psychiatric hospital admissions (to all types of wards) of 6250 CYP (up to 18 years of age) patients in England. About a third of these admissions (37%) were to private hospitals. The mean age of these CYP at their first admission during the follow-up period was 15.3 years (s.d.: 1.7); 70% were female and 18% from BAME backgrounds (Table 1). Where data were available, 11% (of the 86% for whom data were available) were looked-after children and 43% (of the 65% for whom data were available) were in full-time education. The mean number of admissions per person over the 3.25 years was 1.7 (s.d.: 1.2) with an average length of stay per admission of 93 (s.d.: 94) days. The mean number of out-of-area hospital admissions was 0.48 (s.d.: 0.80), reflecting 28% of all admissions.

**Table 1:** Patient characteristics in the entire sample (*n* = 6250)

Variable	Pre-pandemic (*n* = 6156)	Post-pandemic (*n* = 94)	Total sample (*n* = 6250)
Mean age at first admission (s.d.) [median; IQR]	15.3 (1.7) [16;3]	15.6 (1.6) [16;2]	15.3 (1.7) [16;3]
Gender			
Female	70%	72%	70%
Missing	1%	2%	1%
BAME background			
Yes	18%	18%	18%
Missing	7%	6%	7%
Looked after			
Yes	11%	8%	11%
Missing	14%	13%	14%
In full education			
Yes	43%	34%	43%
Missing	35%	47%	35%
Mean number of admissions per patient (s.d.) [median; IQR]	1.7 (1.2) [1;1]	1.2 (0.6) [1;0]	1.7 (1.2) [1;1]
Mean length of stay (s.d.) [median; IQR]	93 (94) [68;94] 6065	65 (65) [43;77] 71	93 (94) [67;94] 6136
Mean number of out-of-area admissions per patient (s.d.) [median; IQR]	0.48 (0.8) [0;1]	0.33 (0.6) [0;1]	0.48 (0.80) [0;1]

s.d.: standard deviation; IQR: Interquartile range expressed as the difference between percentile 75 and percentile 25.

### Impact of the COVID-19 pandemic on hospital admissions

Table 2 shows the results from the ITSA. The level that the trend of each outcome variable started at the beginning of the follow-up period (i.e. January 2018) is presented in column F and the trends before the pandemic in column A. Columns B and C present the immediate impact (i.e. in the first week) and the subsequent trend of lockdown 1 compared with pre-COVID-19 trends. Similarly, columns D and E show the immediate and subsequent trends for lockdown 2. The number of lags used in each ITSA model are presented in column G, the post-lockdown 1 trend in column H (i.e. which is the sum of the trends in columns A and C) and the post-lockdown 2 trend in column I (which is the sum of the trends in columns A, C and E).

**Table 2:** Impact of COVID-19 pandemic in England on child and adolescent mental health services

Outcome variable	Pre-pandemic trend coefficient (s.e.) [95% CI]	Immediate effect (i.e. in the first week) (lockdown 1) coefficient (s.e.) [95% CI]	Subsequent trend (post pandemic) (lockdown 1) coefficient (s.e.) [95% CI]	Immediate effect (lockdown 2) coefficient (s.e.) [95% CI]	Subsequent trend (post pandemic) (lockdown 2) coefficient (s.e.) [95% CI]	Starting level of the trend at the beginning of the follow-up period (i.e. Jan 2018) Constant coefficient (s.e.) [95% CI]	Number of lags used in each ITSA model	Overall post-lockdown trend (lockdown 1) coefficient (s.e.) [95% CI]	Overall post-lockdown trend (lockdown 2) coefficient (s.e.) [95% CI]
Weekly admissions	0.01 (0.13)	**−53.47 (6.58)**	**−0.47 (0.18)**	−1.14 (1.73)	0.25 (0.13)	82.82 (9.95)	5	**−0.46 (0.10)**	**−0.21 (0.09)**
	[−0.26;0.27]	**[−66.47;−40.77]**	**[−0.83;−0.11]**	[−4.55;2.28]	[−0.01;0.50]	[63.17;102.47]		**[−0.65;−0.27]**	**[−0.38;−0.04]**
LOS	−0.40 (0.14)	−2.43 (8.02)	**−0.72 (0.25)**	15.63 (10.04)	−2.59 (1.35)	73.83 (195.27)	6	**−1.12 (0.30)**	**−3.72 (1.39)**
	[−0.68;−0.12]	[−18.27;13.42]	**[−1.22;−0.22]**	[−4.21;35.46]	[−5.26;0.07]	[−311.90;459.57]		**[−1.73;−0.52]**	**[−6.46;−0.97]**
Number of admissions per patient	0.01 (>0.00)	**0.57 (0.14)**	0.01 (0.01)	−0.03 (0.02)	−0.04 (0.02)	1.97 (0.06)	1	0.01 (>0.01)	−0.03 (0.02)
	[0.01;0.01]	**[0.30;0.84]**	[−0.01;0.2]	[−0.08;>0.00]	[−0.08;>0.00]	[1.86;2.08]		[<−0.00;0.03]	[−0.06;0.01]
Percentage of private providers	−0.001 (0.000)	**0.092 (0.020)**	−0.001 (0.001)	−**0.115 (0.039)**	**0.009 (0.003)**	0.426 (0.012)	10	−0.002 (0.001)	**0.007 (0.003)**
	[−0.001;−0.001]	**[0.052;0.131]**	[−0.003;0.002]	**[−0.192;−0.037]**	**[0.002;0.015]**	[0.403;0.450]		[−0.004;0.001]	**[0.002;0.013]**
Percentage of out-of-area admissions (natural clinical flow)	<−0.000 (<0.000)	**0.064 (0.023)**	−0.003 (0.002)	−0.030 (0.037)	0.003 (0.003)	0.228 (0.011)	7	−0.003 (0.002)	0.006 (0.002)
	[<−0.001;>0.000]	**[0.018;0.110**]	[−0.006;0.001]	[−0.102;0.425]	[−0.003;0.010]	[0.205;0.250]		[−0.006;0.006]	[−0.004;0.005]
Percentage of out-of-area admissions (>50 m)	<−0.000 (<0.000)	**0.087 (0.30)**	−0.003 (0.002)	0.019 (0.071)	0.002 (0.004)	0.229 (0.009)	3	−0.003 (0.002)	−0.001 (0.004)
	[−0.001;<−0.001]	**[0.027;0.148]**	[−0.006;0.001]	[−0.121;0.160]	[−0.005;0.009]	[0.210;0.248]		[−0.007;0.001]	[−0.008;0.006]
Age	0.002 (0.001)	**0.545 (0.074)**	−0.009 (0.005)	**0.384 (0.195)**	−0.003 (0.016)	15.29 (0.04)	0	−0.071 (0.001)	−0.010 (0.015)
	[0.001;0.003]	**[0.399;0.690]**	[−0.020;0.002]	**[<0.001;0.769]**	[−0.035;0.028]	[15.21;15.38]		[−0.018;0.004]	[−0.040;0.019]
Percentage, females	<0.000 (<0.000)	0.007 (0.029)	0.003 (0.002)	−0.017 (0.066)	−0.009 (0.005)	0.738 (0.012)	0	**0.003 (0.002)**	−0.006 (0.005)
	[<−0.001;<0.00]	[−0.050;0.063]	[<−0.001;0.006]	[−0.147;0.112]	[−0.019;0.001]	[0.714;0.763]		**[<0.001;0.007]**	[−0.015;0.004]
Percentage, BAME	<0.000 (<0.000)	−0.030 (0.040)	−0.001 (0.002)	0.005 (0.036)	0.005 (0.003)	0.178 (0.005)	15	−0.001 (0.002)	**0.004 (0.002)**
	[<−0.001;<0.00]	[−0.108;0.049]	[−0.005;0.003]	[−0.067;0.076]	[−0.001;0.011]	[0.167;0.189]		[−0.005;0.003]	**[0.001;0.008]**
Percentage, looked-after children	<0.000 (<0.000)	0.035 (0.027)	−0.002 (0.001)	**0.174 (0.030)**	**−0.009 (0.002)**	0.141 (0.010)	10	−0.002 (0.001)	**−0.011 (0.001)**
	[<−0.001;<0.00]	[−0.019;0.089]	[−0.005;0.001]	**[0.116;0.233]**	**[−0.013;−0.005]**	[0.122;0.160]		[−0.005;0.001]	**[−0.013;−0.008]**
Percentage, Q1 IMD	0.000 (0.000)	**−0.041 (0.014)**	−0.001 (0.001)	**0.073 (0.031)**	**−0.005 (0.002)**	0.162 (0.007)	6	<0.001 (0.001)	**−0.006 (0.002)**
	[0.000;0.000]	**[−0.068;−0.013]**	[−0.002;0.001]	**[0.012;0.134]**	**[−0.009;−0.001]**	[0.148;0.176]		[−0.002;0.001]	**(−0.009;−0.002)**

The coefficients of the OLS regression with Newey–West standard errors (s.e.) and 95% confidence intervals (CI). ITSA, interrupted time series analysis; OLS, ordinary least squares; LOS, length of stay; BAME, Black Asian and Minority Ethnic group; Q1, first quintile; IMD, Index of Multiple Deprivation. Statistically significant results of interest are presented in bold.

Prior to the pandemic, there was an average of approximately 83 admissions per week. As Table 2 shows, there were 53.47 (95%CI: −66.47;−40.77) fewer admissions during the first week of the pandemic restrictions, followed by a decrease in weekly trend of hospital admissions relative to the pre-pandemic trend of −0.47 (95%CI: −0.83;−0.11) admissions per week. The weekly number of admissions decreased by −0.46 (95%CI: −0.65;−0.27) (or 2%) after lockdown 1 and by −0.21 (95%CI:−0.38;−0.04) after lockdown 2. Similarly, the average length of stay per admission decreased by 1.12 (95%CI: −1.73;−0.52) days per week after lockdown 1 and by 3.72 (95%CI: −6.46;−0.97) days per week after lockdown 2.

The patients admitted to hospital during the first week of lockdown 1 had on average 0.57 (95%CI: 0.30;0.84) more admissions over the follow-up period, suggesting that during this week more complex cases were admitted to hospital. However, there was no further impact on the trends of the number of admissions per person.

The results also showed an increase in admissions to private hospitals in the first week of lockdown 1 by 9.2 (95%CI: 5.2;13.1) percentage points (p.p), but the system seemed to adjust to pre-pandemic levels after lockdown 2 (i.e. a rapid fall in private admissions by 11.5[th]p.p. (95%CI: 19.2;3.7) followed by a slight increase in weekly trends after lockdown 2).

Out-of-area hospital admissions also increased in the first week of lockdown 1 by 6.4[th]p.p. (95%CI: 1.8;11.0), as indicated by the clinicians’ defined natural clinical flow, and 8.7[th]p.p. (95%CI: 2.7;14.8) based on distance (i.e. >[th]50 miles). However, there was no further impact on the level or trends of out-of-area admissions.

In terms of changes in the sociodemographic characteristics of CYP admitted to hospital, the mean age was 0.545 (95%CI: 0.399;0.690) years greater during the first week of lockdown 1 and increased by another 0.384 (95%CI: <0.001;0.769) years during the first week of lockdown 2.

There was an increase of 0.3[th]p.p. (95%CI: <0.1;0.7) per week in the proportion of females admitted after lockdown 1 and 0.4[th]p.p. (95%CI: 0.1;0.8) per week in the proportion of patients from BAME backgrounds admitted after lockdown 2. After lockdown 2, there was an immediate (i.e. in the first week of the lockdown) increase in the proportion of looked-after children admitted to hospital by 17.4[th]p.p. (95%CI: 11.6;1.3) followed by a downward trend of 1.1 [th]p.p. (95%CI: 0.8;1.3).

Similarly, there was a downward trend in the admission of patients from the most socioeconomically deprived areas by 0.6[th]p.p. (95%CI: 0.2;0.9) per week after lockdown 2. This trend could have reflected a downward adjustment after a considerable increase in the admissions of patients from the most socioeconomically deprived areas by 7.3[th]pp (95%CI: 1.2;13.4) during the first week of lockdown 2.

**Fig. 1:**
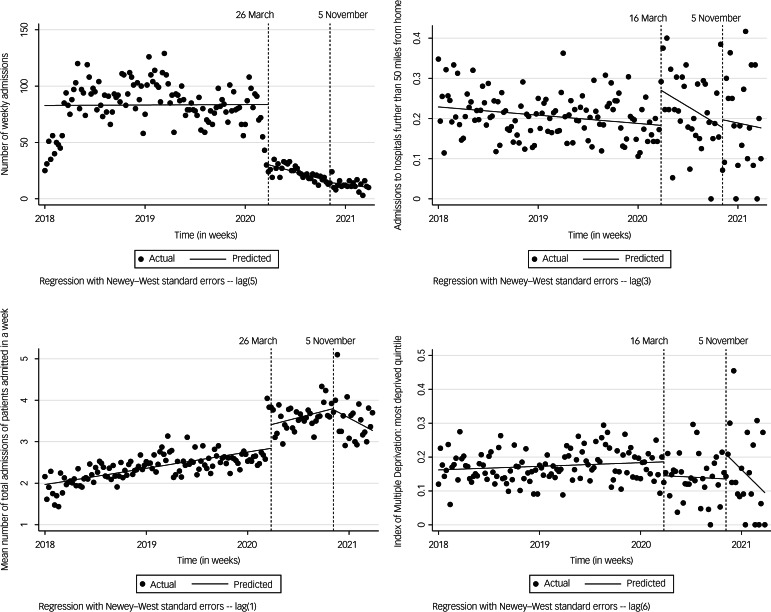
Interrupted time-series graphs ofkey outcomes.

Figure 1 presents graphically the impact of the two lockdowns on the trends of four main outcome variables. The upper left graph of Fig. 1 shows a considerable (65%) decrease in admissions immediately after lockdown 1. The upper right graph depicts the trends in out-of-area admissions, with a very rapid increase followed by a slower decrease back to pre-pandemic levels. The lower left graph depicts the trends in the mean number of total admissions of people admitted in a week. The lower right graph shows contrasting findings in admissions amongst CYP from the most deprived quintile across the two lockdowns. A fall in admissions was noted immediately after lockdown 1, but the trend was not sustained, and the percentage of admissions involving CYP from the most deprived areas went back to pre-pandemic levels. On the other hand, although immediately after lockdown 2, CYP from the most deprived quintile experienced a significant rise in admissions, this rise was followed by a minimal but sustained decrease in admission trends.

## Discussion

The COVID-19 pandemic had a significant impact on the functioning of mental health services and the use of in-patient beds within England. To our knowledge, this is the first national-level study investigating the impact of COVID-19 and related public health measures on CYP hospital in-patient admissions during the pandemic across England.

These national data indicate that across England there was a significant decrease in hospital admissions during lockdown 1 and a further decrease in lockdown 2. This is likely to be because of a raising of the threshold for admission referral and acceptance, and prioritisation of the most unwell to be admitted to hospital, as there were staffing shortages and bed closures during that period. This is supported by our findings that during the early part of lockdown 1, there was a higher number of hospital admissions during the follow-up period compared to those admitted pre-pandemic, indicating a greater severity of illness. Alternatively, it could also be possible that there was increased community provision of mental health services, including alternative modes of service delivery (i.e. online appointments); however, national service data suggested that a decrease of community referrals was observed during the same period.^12^

Although the overall number of admissions fell at the beginning of lockdown, a significantly higher percentage of those who were admitted went to private sector units. These findings may be the result of government policies to engage with independent sector providers during the unprecedented redeployment of NHS resources to manage COVID-19, or differences in agility in changing practice in response to restrictions between the NHS and the private sector. Outsourcing of NHS services to the private sector is increasingly common in England,^13^ and this may have increased during the pandemic for some services including psychiatric in-patient services.

There was also an initial increase in the number of out-of-area admissions during lockdown. Out-of-area admissions have been an ongoing problem within Child and Adolescent Mental Health Services (CAMHS), even prior to the pandemic.^6^ It is concerning to have seen a rise in out-of-area admissions during COVID-19, because it is likely that the combination of the distance, the risks of contracting and spreading the virus, and government restrictions will have had added to the complexity of these admissions. Following the initial rise in out-of-area admissions, these fell across the lockdowns to return to pre-pandemic levels; however, many would argue that these levels should be lower than they currently are and that services should be better prepared to avoid out-of-area admissions in such a vulnerable population.

The mean length of hospital stay was steadily decreasing prior to the pandemic. However, the length of stay fell more rapidly during the pandemic. The final phase of a psychiatric admission usually includes regular periods of home leave of increasing length, until clinicians are satisfied that the patient is safe to be discharged. However, since the locations to which an individual could go on leave were restricted, and any time at home counted as an infection risk which necessitated repeated testing and quarantining prior to returning to the ward, this process was often not feasible. Clinicians were therefore required to discharge individuals with few to no trial periods of leave. This necessary change in practice may also explain the higher-than-normal readmission rate during this period.

There was a reduction in the proportion of admitted males during lockdowns 1 and 2. The reasons for this are not clear and may represent a lower amount of help-seeking amongst males or overall lower self-harming or risk behaviours. Lockdowns may also have restricted access to alcohol and illicit substances, which are overall used more frequently by young males compared to females,^14^ and can have negative effects on mental health and risk within young people.^15^

There was a significant rise in admissions of those from the most deprived areas of the country at the start of lockdown 1. Studies have shown that the COVID-19 pandemic most significantly impacted those from deprived areas,^16^ and these findings suggest that it worsened health inequalities within child and adolescent mental healthcare.

### Comparison with other studies

Studies have indicated that the COVID-19 pandemic had a substantial impact on public mental health and that certain population groups were at greater risk of worsening mental health. Chen et al (2020) looked at medium-term trends in secondary care psychiatry referrals and found that across the population, there was an initial fall in referrals followed by an acceleration in the referral rate compared to the previous year^17^ However, when groups suspected to be more vulnerable to the effects of the pandemic were analysed separately, such as older adults and CYP, this trend was not seen.^18^ This fits with our data since there is no rapid re-increase in admissions following the initial fall; instead, there were further decreases in admission numbers. Bakolis et al (2020) also found ongoing reduced caseloads for CAMHS both during and after lockdown 1, with more non face-to-face contacts.^19^ Our finding of higher complexity of the cases who did present during lockdown is also in line with the findings of Mukadam et al (2021)who showed lower numbers of psychiatric presentations to emergency departments, but of those who presented, a higher proportion were admitted to in-patient units.^20^ Steeg et al^21^ reviewed studies of self-harm presentations finding sustained reductions in service utilisation during 2020, which correlates with our findings; however, there were increases in service utilisation following self-harm in adolescents from 2021.^22^

### Limitations of this study

The main strength of this study is the use of individual patient data from the entire country that ensures the generalisability of the results across England. However, it is also subject to limitations. First, this routinely collected data did not capture information about young people who may have been close to the threshold for admission but were not admitted. Second, it was not possible to fully disentangle the impact of the pandemic on the population's mental health from the impact of policy responses on the provision of in-patient care. However, it is important to analyse and reflect upon the events of the COVID-19 pandemic in order for the NHS to devise and navigate recovery plans following the pandemic, as well as optimising any future pandemic responses. Last, we did not investigate the impact of the pandemic on out-patient visits, primary care presentations or emergency department presentations. Studies have found a decreased total number of presentations to hospital emergency departments, and increased proportion of children with self-harm presentations, but no increase in the proportion of severe self-harm within those presenting with self-harm.^22^

### Service implications

There is consensus that the pandemic resulted in significant implications for CYP mental health, including an increased burden of poor mental health and potentially substantial demand for services.^5^ However, specific subgroups may have experienced greater risks and difficulties, such as CYP living in deprived areas, those from BAME backgrounds, and vulnerable children, including looked-after children. To avoid deepening or widening inequalities, it will be necessary to not only actively identify the extent of these inequalities and associated drivers but to also reach out to these populations to address hidden, unmet needs and provide a universally proportionate response.^21^

With the phased delegation of specialised mental health services to the Integrated Care Systems beginning in July 2022, the commissioning of CYP mental health services in England is moving away from the historic four-tier structural framework of service provision to unspecialised, universal services: tier one funded by local authorities, tiers two to three funded by clinical commissioning groups, and in-patient treatment facilities at tier four funded by NHS specialised commissioning teams. However, the pre-existing variation in commissioning and delivery structures has led to a complex, fragmented system with variability in the quality of patient outcomes.^23,24^

The key findings of our study, reflecting decreased hospital admissions during the pandemic along with a concurrent decrease in community provision as per the publicly available data on community provision, highlights the importance of focusing on expanding the provision of preventative and community-based services, ensuring equitable access, and the potential of preventing much longer, more expensive courses of in-patient treatment for repeated or complex admissions.

Similar to other studies,^25^ we recommend the following to inform commissioning while accounting for the direct and indirect impact of the ongoing COVID-19 pandemic:

- Map the existing provision of CYP mental health services along with system-level understanding of funding including system-developmental monies across all tiers of the CYP pathway, to enable accurate estimates of treatment gaps and effective commissioning of services.
- Working with local authorities, integrated care systems should maximise the prevention offer of early childhood services while expanding and improving quality, provision and access associated with low-level, preventative and universal CYP mental health services.
- Identify avoidable health inequalities alongside risk factors (including protective factors) across the CYP mental health pathway.

## Data Availability

The data that support this study were obtained from the national specialised mental health Patient Level Dataset that prohibits using or sharing the data beyond this study.

## Reporting statement

The RECORD statement was used to report the methods and results of the study.^26^
